# Calorie restriction mimetic drugs could favorably influence gut microbiota leading to lifespan extension

**DOI:** 10.1007/s11357-023-00851-0

**Published:** 2023-06-30

**Authors:** Tomoya Shintani, Hideya Shintani, Masashi Sato, Hisashi Ashida

**Affiliations:** 1 Graduate School of Science, Technology and Innovation, Kobe University, 1-1 Rokkodai-Cho, Nada, Kobe, Hyogo 657-8501 Japan; 2 The Japanese Clinical Nutrition Association, 2-16-28 Ohashi, Meguro, Tokyo 153-0044 Japan; 3 Department of Internal Medicine, Towa Hospital, 4-13-15 Tanabe, Higashisumiyoshi, Osaka 546-0031 Japan; 4 Department of Internal Medicine, Osaka Saiseikai Izuo Hospital, 3-4-5 Kitamura, Taisho, Osaka 551-0032 Japan; 5 Faculty of Agriculture, Kagawa University, 2393 Ikenobe, Miki, Kagawa 761-0701 Japan; 6 Faculty of Biology-Oriented Science and Technology, Kindai University, 930 Nishimitani, Kinokawa, Wakayama 649-6493 Japan

**Keywords:** Calorie restriction mimetics, Microbiome, Anti-aging, Acarbose, Glucosamine

## Abstract

Calorie restriction (CR) can prolong human lifespan, but enforcing long-term CR is difficult. Thus, a drug that reproduces the effects of CR without CR is required. More than 10 drugs have been listed as CR mimetics (CRM), and some of which are conventionally categorized as upstream-type CRMs showing glycolytic inhibition, whereas the others are categorized as downstream-type CRMs that regulate or genetically modulate intracellular signaling proteins. Intriguingly, recent reports have revealed the beneficial effects of CRMs on the body such as improving the host body condition via intestinal bacteria and their metabolites. This beneficial effect of gut microbiota may lead to lifespan extension. Thus, CRMs may have a dual effect on longevity. However, no reports have collectively discussed them as CRMs; hence, our knowledge about CRM and its physiological effects on the host remains fragmentary. This study is the first to present and collectively discuss the accumulative evidence of CRMs improving the gut environments for healthy lifespan extension, after enumerating the latest scientific findings related to the gut microbiome and CR. The conclusion drawn from this discussion is that CRM may partially extend the lifespan through its effect on the gut microbiota. CRMs increase beneficial bacteria abundance by decreasing harmful bacteria rather than increasing the diversity of the microbiome. Thus, the effect of CRMs on the gut could be different from that of conventional prebiotics and seemed similar to that of next-generation prebiotics.

## Introduction

Research on medicine and nutrition is often intended to maintain and promote health, ultimately leading to a healthy aging society. In research on aging, only the dietary regimen for longevity has gained remarkable consensus. Calorie restriction (CR) is the most common method used for healthy aging [1, 2]. CR is a dietary regimen that reduces calorie intake without causing malnutrition [3]. CR is sometimes used to control body weight and improve health and quality of life [4]. Comprehensive Assessment of Long-Term Effects of Reducing Intake of Energy (CALERIE) trials are being conducted to test the effects of CR on aging- and longevity-related outcomes in humans [5]. Designed from CALERIE phase 1, CALERIE phase 2 is a large-scale clinical study to assess the effect of sustained CR in healthy humans. The outcomes of the 2-year randomized controlled trial comprising over 200 participants showed that moderate CR induced improvements in aging-related biomarkers [6]. Thus, it seems likely that CR could prolong human lifespan. However, enforcing long-term CR is difficult in terms of the quality of life [7]. Therefore, a drug that reproduces the effects of CR without CR is required.

**Fig. 1:**
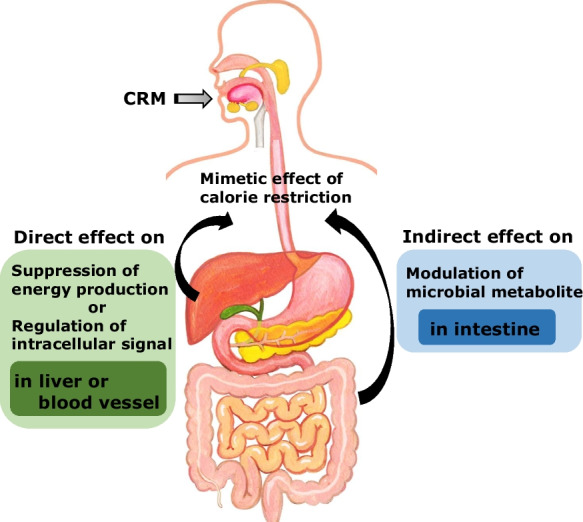
The concept of dual effects of CRMs. The dual effects are direct effect on metabolism of glucose and lipid in mainly liver or blood vessel and indirect effect on modulation of microbial metabolite in intestine

The widely accepted definition of CR mimetics (CRMs) is compounds that mimic the biochemical and functional effects of CR[8, 9]. The concept of CR mimetics (CRMs) was first proposed in 1998 by Lane et al. [10] in a study of 2-deoxy-D-glucose, which favorably alters aging-related biomarkers in rodents. To date, more than 10 drugs have been listed as CRM in many studies based on the direct effects of numerous compounds on mammalian cells. Some of them are conventionally categorized as upstream-type CRMs that suppress energy production [11], whereas others are categorized as downstream-type CRMs that regulate or genetically modulate intracellular signaling proteins [12]. Among these CRMs, we previously focused on the direct effects of upstream-type CRMs, mainly in the liver or vascular endothelium, and reported that the optimization of glucose metabolism, particularly the enhancement of fat oxidation and moderate production of reactive oxygen species, is the most remarkable characteristic [2]. Intriguingly, recent reports have revealed that CRM compounds can improve the host body condition by utilizing intestinal bacteria and their metabolites. Therefore, CRMs may have dual favorable effects on lifespan. However, our knowledge of the physiological effects of CRM in humans is fragmentary. In the current study, we focused on the indirect effects of CRMs on gut microbes (Fig. 1). This review covered bioactive carbohydrates, such as D-glucosamine, D-allulose, and D-allose, and antidiabetic drugs, such as metformin, acarbose, and sodium-glucose cotransporter 2 inhibitors (SGLT-2)(Table 1). Additionally, we reviewed other promising anti-aging CRMs, such as rapamycin, resveratrol, and polyamines. The compounds discussed in this paper were aimed to be exhaustive, but there are other compounds that were not necessarily included. Notably, 2-deoxy-D-glucose, which was previously mentioned as a first candidate for CRMs, has not been addressed in this review because its cardiotoxicity in rats was confirmed, making its use as a CRM less likely [13].

**Table 1:** Characteristics and targets of CRMs

CRMs	Main characteristics	Types of CRMs	Target of the direct effect as a CRM
Metformin	Anti-diabetic drug	Downstream	Intracellular energy sensor activation
Acarbose	Anti-diabetic drug	Upstream	Intestinal glycosidase inhibition
SGLT2 inhibitor	Anti-diabetic drug	Upstream	Glucose excretion
D-Glucosamine	Dietary supplement	Upstream	Glycolysis adjustment
D-Allulose	Food ingredients	Upstream	Glycolysis improvement
D-Allose	Food ingredients	Upstream	Glucose metabolism reduction
Resveratrol	Wine polyphenol	Downstream	Longevity gene activation
Rapamycin	Immunosuppressant drug	Downstream	Amino acid sensor inhibition
Polyamines	Gut bacterial metabolite	Downstream	Epigenetic control

This paper introduces the latest information and scientific basis for research on aging and intestinal bacteria. Next, we summarize the functionality and characteristics of each CRM compound. Finally, we, for the first time, discuss the effects of CRM on gut bacteria and the prospective studies.

## Diet, gut microbe, and aging

The human intestinal tract is composed of a considerable microbiota population that lives symbiotically within the host. Recently, awareness of the importance of microbial communities in human health has increased tremendously, resulting in the science of microbiome evolving as an important area for biomedical sciences [14]. Gut microbial flora belong to four main phyla: Bacillota (formerly Firmicutes), Bacteroidota (formerly Bacteroidetes), Actinomycetota (formerly Actinobacteria), and Pseudomonadota (formerly Proteobacteria) [15]. In addition to these four major phyla, the human gut microbiota often includes the phylum Verrucomicrobia [16], although its relative abundance is low. The balance among colonizing species and conditions in the intestines influence overall health [17]. Maintaining a good microbiota balance and a rich abundance of Actinomycetota is expected to support a healthy intestinal environment [18].

Some gut microbe groups produce organic acids, specifically short-chain fatty acids (SCFAs). Increased intestinal SCFAs are often considered a positive outcome because they play important roles in gut health and overall health [19, 20]. SCFAs are produced by gut bacteria as they ferment dietary fiber and other complex carbohydrates [21]. These compounds have been shown to have several beneficial effects on the gut and the body, including the next four items. First is providing energy. SCFAs can be used as an energy source by intestinal cells and other cells in the body [22]. Second is promoting gut health. SCFAs help to maintain a healthy gut environment by regulating the pH, promoting the growth of beneficial bacteria, and inhibiting the growth of harmful bacteria [23]. Third is reducing inflammation: SCFAs have been shown to have anti-inflammatory effects in the gut and the body, which may help to reduce the risk of chronic diseases such as inflammatory bowel disease and colon cancer [24]. Fourth is regulating metabolism: SCFAs have been shown to play a role in regulating metabolism and may help to improve insulin sensitivity and reduce the risk of type 2 diabetes [25]. Therefore, increased production of SCFAs can be a positive outcome, as it is often associated with improved gut health and overall health. However, it is important to note that the specific effects of SCFAs may vary depending on the type and amount of SCFAs produced, as well as the individual's diet and gut microbiota composition.

Various factors including age, living environment, birth delivery route, breastfeeding, antibiotics, prescribed medicines and dietary conditions, and exercise influence gut microbial composition and function [26]. This mentioned several factors should list others not included. Intriguingly, the intestinal microbiota changes gradually with age[18]. The relative abundance of *Bifidobacterium* species, which includes beneficial bacteria of the phylum Actinomycetota, decreases with age [18]. Bacteroidota species influence body weight maintenance and intestinal immunity [27, 28]. Beneficial bacteria in the Actinomycetota and Bacteroidetes phyla produce SCFAs that improve the intestinal environment and help maintain good health [29]. However, the relative abundance of bacteria in the phylum Bacillota appeared to be associated with obesity [30]. Thus, the Bacillota/Bacteroidetes ratio is known to increase obesity [31]. Interestingly, this ratio is positively associated to some extent with aging [31]. A similar phenomenon to that observed in humans, where the intestinal microbiota changes due to aging, has also been observed in mice [32]. Note that the data from preclinical studies have been addressed in this review. The microbiome in gut of extremely old people (individuals who are over 100 years of age), even accommodating opportunistic bacteria, is reported to be enriched in *Akkermansia* belonging to the phylum Verrucomicrobia [33]. As a side note, the mentioned opportunistic bacteria are a type of bacteria that can cause infections in people who have weakened immune systems, whose examples of these main opportunistic bacteria were some groups in Bacteroidetes and Enterobacteriaceae group in Pseudomonadota.

Studies in humans have revealed that dietary conditions contribute to gut microbes [26]. Recently, CR diets, especially carbohydrate-restricted diets, have been confirmed to differentially alter the composition of gut microbiota when compared with the effect of high-fat diets. Furthermore, only CR diets were able to provide positive gut-associated systemic outcomes [34]. The study found that a ketogenic diet alters the gut microbiome, leading to a decrease in intestinal Th17 cells, a type of immune cell that plays a role in inflammatory responses. The authors suggested that this may be a mechanism underlying the observed health benefits of ketogenic diets, which have been shown to improve glucose regulation and reduce inflammation. The study also showed that a restricted diet positively affected the gut ecosystem through a mechanism involving the concomitant host production of intestinal organic acids [34]. Additionally, the interplay between the restricted diet and microbiota plays a pivotal role in manifesting the beneficial effects of restricted diet [35]. CR increased Bacteroidetes and significantly reduced the Bacillota/Bacteroidota ratio in obese mice [36]. In young humans, long-term CR also reduces the Bacillota/Bacteroidota ratio[37]. CR enhanced the growth of beneficial microorganisms such as *Bacteroides*, *Roseburia*, *Faecalibacterium*, and *Clostridium* XIVa. The mechanism on the efficacy of CR might be related with the result of recent study on fasting in mice [38]. The expression of bile acid metabolism-related genes in the liver and the ileum was reported to decrease in the fasting mice, who have more of *Akkermansia* and *Parabacteroides*.

## Effects of metformin, acarbose, and SGLT-2 inhibitor on gut microbe

### Metformin

**Fig. 2:**
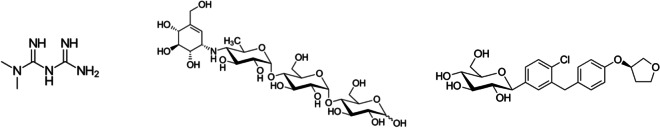
The molecular structures of metformin (left), acarbose (middle), and SGLT2 inhibitor (empagliflozin) (right) were shown

Metformin (Fig. 2) is the most prescribed drug worldwide for the management of diabetes, either alone or in combination with insulin or other hypoglycemic therapies[39]. It has few serious side effects, but the most common side effect is gastrointestinal issues such as nausea, vomiting, and diarrhea [25, 40]. Metformin can also cause liver dysfunction, vitamin B12 deficiency, lactic acidosis, hypoglycemia, and skin reactions [41-43]. However, most people who take metformin do not experience significant side effects, and the benefits of the medication often outweigh the risks.

Interestingly, metformin has attracted attention as a potential CRM [2, 44]. As a CRM, the direct effects of metformin are mediated by AMP-activated protein kinase (AMPK) [45]. Metformin transiently inhibits the mitochondrial respiratory chain, increases the intracellular AMP/ATP ratio, and activates AMPK, leading to improved glucose metabolism [46]. A novel pathway for metformin to excrete glucose into the intestinal tract has been reported [47]. Thus, metformin exerts its effect on the intestinal flora by changing the level of carbohydrates that entered into cecum. Several interesting reports have been published regarding the action of metformin in the intestine [48].

An increase in the *Akkermansia* population induced by metformin treatment has been reported to improve glucose homeostasis in mice with diet-induced obesity [49]. Metformin might also increase ursodeoxycholic acid levels by reducing the relative abundance of *Bacteroides fragilis* in the large intestine and favorably alter glucose tolerance via intestinal farnesoid X receptor signaling [50].

A clinical trial showed that an increase in the Bacillota/Bacteroidota ratio is related to low-grade inflammation and increased capability to harvest energy from food [51]. A small-scale clinical trial reported that on one hand, the relative abundance of *Intestinibacter* and *Clostridium* decreased [52]; on the other hand, the relative abundance of *Escherichia*/*Shigella* and *Bilophila wadsworthia* increased. A meta-analysis showed that oral metformin might induce selective growth of *Escherichia coli* and upregulate the secretion of SCFAs, ultimately contributing to improve insulin sensitivity [53].

### Acarbose

Acarbose (Fig. 2) is an α-glycosidase inhibitor that delays the digestion of carbohydrates into absorbable monosaccharides, thereby reducing the postprandial blood glucose peak [54]. The most common side effect of acarbose is gastrointestinal issues such as bloating, gas, abdominal pain, and diarrhea [55, 56]. Acarbose can also cause hypoglycemia, elevated liver enzymes, allergic reactions, and interference with digestion [57, 58], although many people who take acarbose do not experience significant side effects.

This antidiabetic drug significantly increased the median lifespan of male mice by 22% [59]. However, acarbose causes bloating as a side effect [55] when the carbohydrate that were not digested by acarbose, such as starch, enter the large intestine [60]. In addition to reducing the absorption of glucose derived from starch, inhibition of host digestive enzymes by acarbose results in increased flow of polysaccharide substrate to the lower digestive system [7], approximately mimicking the efficacy of resistant carbohydrate consumption in the colon. In fact, acarbose has been shown to increase the concentration of non-digested carbohydrates in stool [61] and the observed increased excretion of hydrogen in breath, which is a result of fermentation by the gut microbiota [62]. Thus, acarbose is expected to change gut microbe profiles and conditions. Interestingly, a shotgun metagenomic sequencing of fecal samples from approximately 4200 patients, showed that α-glucosidase inhibitors had the strongest effect on the intestinal microbiota among a total of 759 drugs, except for gastrointestinal medications [63].

Changes in the gut microbiome and fermentation products were concurrent with enhanced longevity in acarbose-treated mice [64]. Acarbose-treated mice exhibited decreased fecal bacterial diversity. The Chao1 richness estimate decreased from 229 in the control mice to 199 in the acarbose-treated mice. Simpson’s evenness—another index of microbial diversity—was also lower in acarbose-treated mice than that in untreated mice. The relative abundance of Muribaculaceae increased, whereas those of Lactobacillaceae and Erysipelotrichaceae decreased.

In randomized controlled clinical trials with prediabetic patients, acarbose has been reported to alter the intestinal bacteria [65]. The diversity of the gut microbes did not change. Lactobacillaceae, Ruminococcaceae, and Veillonellaceae were enriched by acarbose. In contrast, Ruminococcaceae and Lachnospiraceae abundance decreased.

### SGLT-2 inhibitor

Sodium-glucose cotransporter 2 (SGLT2) inhibitors (Fig. 2) are a class of drugs traditionally used to treat diabetes. Currently, they are also indicated for chronic heart failure and chronic renal failure. SGLT-2 inhibitors include canagliflozin, dapagliflozin, and empagliflozin, which have been approved for use in adults. Common side effects of SGLT-2 inhibitors include genital and urinary tract infections, hypoglycemia, dehydration, normoglycemic ketoacidosis, bone fractures, and ketoacidosis [66, 67]. However, the benefits of SGLT-2 inhibitors often outweigh the risks.

Their mechanism of action involves the inhibition of SGLT-2 in the proximal renal tubules and promotion of urinary glucose excretion by inhibiting glucose reabsorption [68]. This mechanism of action not only reduces plasma glucose but also has other beneficial effects, such as weight loss and lowering of blood pressure [69]. However, contrary to expectations, the side effects may be attributed to SGLT-2-mediated inhibition of SGLT-1, which enables glucose absorption in the intestinal tract. Indeed, in mice with renal failure, inhibition of SGLT-1, which aids glucose absorption in the small intestinal epithelium, has been effective in reducing the levels of the urinary toxin phenyl sulfate, derived from intestinal bacteria, in blood [70]. Thus, inhibition of intestinal SGLT1 influences the gut environment. Actually, some effects of SGLT-2 inhibitors on intestinal bacteria have been previously reported, as expanded on below.

Empagliflozin, an SGLT-2 inhibitor, has been reported to alter the intestinal bacteria in C57BL/6 mice [71]. The abundance of organic acid-producing bacteria *Bacteroides* and *Odoribacter* increased, whereas that of the harmful bacteria *Oscillibacter*, which is involved in inflammation, decreased. In another preclinical study, canagliflozin significantly increased short-chain fatty acids in a mouse model of kidney disease, suggesting the promotion of bacterial carbohydrate fermentation in the intestine [72]. In addition, canagliflozin significantly and favorably altered the microbiota composition in mice. The abundance of Actinobacteria increased with canagliflozin treatment. The relative abundance of *Bifidobacterium* increased, whereas that of *Oscillospira* decreased. *Oscillospira* is enriched in lean subjects and decreases with the incidence of inflammatory diseases [73].

SGLT-2 inhibitors have been reported to alter intestinal bacteria in clinical trials [74]. Empagliflozin alters the gut microbiota. Empagliflozin increased sphingomyelin levels but decreased glycochenodeoxycholate, cis-aconitate, and uric acid levels in the blood. Empagliflozin increased the relative abundance of short-chain fatty acid-producing bacteria, such as *Roseburia*, *Eubacterium*, and *Faecalibacterium*, and decreased that of harmful bacteria such as *Escherichia-Shigella*, *Bilophila*, and *Hungatella*.

## Effects of D-glucosamine, D-allulose, and D-allose on gut microbe

### D-Glucosamine

**Fig. 3:**
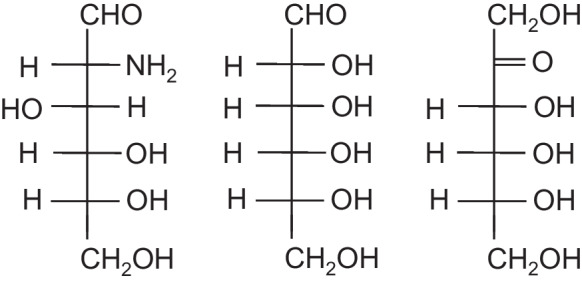
The liner structures of D-glucosamine (left), D-allose (middle), and D-allulose (right) were shown

D-Glucosamine (Fig. 3) is a dietary supplement used to treat osteoarthritis and other joint conditions [75]. The most common side effects of glucosamine include gastrointestinal issues, allergic reactions, and blood sugar changes [76, 77]. However, most people who take glucosamine do not experience significant side effects, and the benefits of the supplement often outweigh the risks.

D-Glucosamine induces autophagy in human cells and prolongs lifespan [78, 79]. A few large epidemiological studies have shown that D-glucosamine could be a promising anti-aging drug [80]. Recently, a Mendelian randomization study revealed that lifelong higher levels of glucosamine may increase life expectancy [81]. However, when D-glucosamine is orally administered, only 44% ingested is absorbed by the intestine [82]. Therefore, the remaining 56% of D-glucosamine possibly influences gut microbes and conditions.

In preclinical trials, D-glucosamine altered intestinal bacteria[83]. This study examined the effect of a 5-month D-glucosamine administration on fecal-microbiome profiles in mice. The α-diversity of the gut microbes and species richness did not change. The relative abundances of several beneficial bacteria in the D-glucosamine group were significantly higher than those in the high-fat diet control group, including that of *Bifidobacterium*, *Akkermansia*, *Lactobacillus*, and *Allobaculum*. Additionally, D-glucosamine treatment suppressed the increase in some harmful bacteria, such as *Roseburia*, *Desulfovibrio*, *Oscillibacter*, and *Intestinimonas*. *Roseburia* is negatively associated with some diseases, including irritable bowel syndrome, obesity, diabetes, and allergies [84]. *Desulfovibrio* belongs to the phylum Proteobacteria and is reported to be involved in autism, Parkinson's disease, and inflammatory bowel diseases [85-87]. In clinical studies, it altered the intestinal microflora [88]. The α-diversity of the bacterial communities in the fecal content was significantly decreased following D-glucosamine intake compared with that before intake. The changes in β-diversity between the samples were not significantly different from the value before intake. The relative abundances of Peptococcaceae and Bacillaceae were also significantly reduced after D-glucosamine intake. D-glucosamine supplementation had no effect on individual or total short-chain fatty acids.

### D-Allulose

D-Allulose (Fig. 3) is a low-calorie sugar substitute that is generally safe for consumption [89], but a few people may experience side effects. The most common side effects of D-allulose include gastrointestinal problems [90]. However, most people who consume D-allulose do not experience significant side effects, and the benefits of the sugar substitute often outweigh the risks.

D-Allulose favorably alters glucose homeostasis via glucokinase and prolongs lifespan via AMPK in animal models [91, 92]. Based on the dynamics of orally administrated D-allulose in body, it is not fully absorbed from the intestine. Approximately 70% of ingested D-allulose is absorbed in the small intestine, and the unabsorbed 30% of ingested D-allulose flows into the large intestine [93]. Thus, the remaining 30% of D-allulose is expected to modulate gut microbes and conditions.

Preclinical trials have reported that D-allulose alters intestinal bacteria [94] by changing the diversity of the gut microbe. The relative abundance of *Lactobacillus*, *Coprococcus*, and *Coprobacillus* increased. *Coprococcus* is the primary butyrate-producing bacterium [95]. In contrast, the relative abundances of *Turicibacter*, Clostridiaceae, *Dorea*, and Erysipelotrichaceae decreased. Another preclinical study showed that D-allulose closely interacted with candidate genes and microbes to alleviate weight gain and inflammation [96]. It also showed that D-allulose increased *Lactobacillus* and *Coprococcus* abundance in the gut microbiota composition [96].

D-Allulose has been shown to alter the intestinal microflora in humans [97]. Intriguingly, *Coprococcus* level was significantly increased, which is supported by multiple preclinical studies. The clinical study was designed for 1-month trial with 15 g of D-allulose intake in 14 participants with slightly higher blood LDL-cholesterol and glucose levels. The results of trial showed that the relative abundance of *Coprococcus* in the intestinal flora increased significantly from 4.2 to 6.4%. *Coprococcus* is known as the main butyrate-producing bacteria [95]. In addition, the abundance of *Blautia* in the gut of volunteers who received D-allulose tended to increase. *Blautia* has beneficial effects on acetic acid production [98]. Thus, D-allulose acts as both a CRM and a potential enhancer for the growth of some specific beneficial intestinal bacteria.

### D-Allose

D-Allose, an isomer of D-allulose (Fig. 3), exerts various beneficial effects such as anti-hypertension, anti-tumor, and protective effects against ischemia–reperfusion [99-101]. D-Allose is generally considered safe for consumption, and there are no known side effects associated with its use. However, some individuals may have a gastrointestinal problem, whose reason is close similarity of D-allose and D-allulose at the molecular structure. There is limited research on the long-term effects of consuming D-allose in large amounts, so it is not clear if there are any potential health risks associated with its use.

Recently, it was reported to prolong life [102, 103]. However, D-allose is not absorbed by the small intestine [104]. Unabsorbed D-allose flows into the large intestine and finally reaches the feces [105]. Thus, D-allose is expected to affect the gut microbiome.

D-Allose has been reported to increase the abundance of *Bacteroides acidifaciens* and *Akkermansia muciniphila* in aged mice[106]. The cecum weights of the control and D-allose groups were similar, although the influence of D-allose on the diversity of mouse gut microbiota has not been reported. In aged mice, the D-allose group increased the relative abundance of Actinomycetota, whereas it decreased that of Pseudomonadota, *Blautia*, and Lachnospiraceae bacteria. D-Allose has not been reported to alter the intestinal microflora in humans.

## Effects of rapamycin, resveratrol, and polyamines on gut microbe

### Rapamycin

**Fig. 4:**
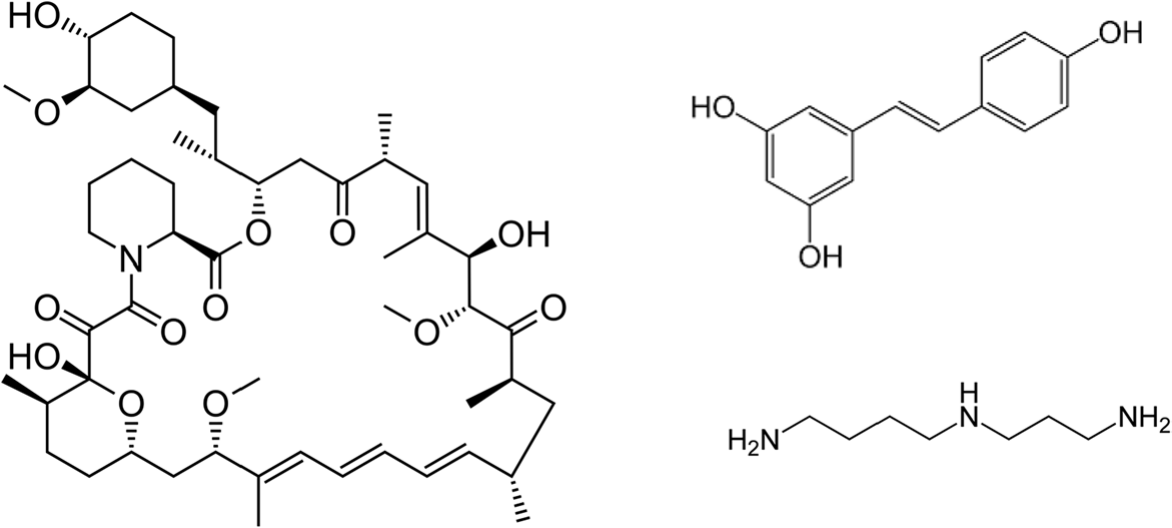
The molecular structures of rapamycin (left), resveratrol (right above), and polyamines (spermidine) (right below) were shown

Rapamycin (Fig. 4) is widely used in biomedical sciences as the inhibitor of the mammalian target of the drug rapamycin (mTOR). Rapamycin is a medication used to prevent organ rejection in organ transplantation or to treat a lymphangioleiomyomatosis [107]. It has potential side effects such as mouth sores, diarrhea, nausea, vomiting, and decline in lung function [108]. It can suppress the immune system, which makes it more difficult to fight off infections.

Rapamycin substantially regulates protein homeostasis, cell proliferation, and inflammation [109]. Rapamycin prolonged the lifespan of adult mice by 30% [110]. Another preclinical study showed that 3 months of rapamycin administration increased the average lifespan and maintained the health of adult mice [111].

In a preclinical study, the relative abundances of Marinilabiliaceae and Turicibacter decreased in response to rapamycin treatment [112]. Rapamycin influenced the relative abundance of *Alloprevotella*, unclassified *Porphyromonadaceae*, *Ruminococcus*, *Bifidobacterium*, *Marvinbryantia*, *Ruminococcus, Helicobacter*, and *Coprobacillus* in mice fed a high-fat diet. In another study, during microbiome analysis, among the most notable changes observed in fecal bacterial DNA content was a significant increase in prevalence of *Candidatus arthromitus* DNA in rapamycin-treated mice [111]. However, a clinical study on the effects of rapamycin on the gut has not been reported.

### Resveratrol

Resveratrol (Fig. 4) is a natural polyphenolic phytoalexin mainly present in red wine [113]. Resveratrol is a compound found in certain plants that can be taken as a dietary supplement. Some potential side effects of resveratrol include gastrointestinal problems [114]. It can also interfere with kidney function and interact with certain medications.

This polyphenol has been thoroughly studied as a compound that activates sirtuin 1 or its invertebrate homologs [115]. Resveratrol protects living organisms against ROS and exerts its antioxidant effects by activating SIRT2 to deacetylate peroxiredoxin 1 [116]. Extension effects on the mean lifespan were observed when resveratrol was administered to obese mice fed a high-fat diet [117]. Resveratrol also preserved indices of vascular function in normal rats but did not extend their lifespan [118].

Resveratrol improved the intestinal microflora imbalance caused by high-fat diet. The mechanisms include reducing the Bacillota/Bacteroidota ratio and promoting the diversity of intestinal microflora by inhibiting the growth of *Enterococcus faecalis* and increasing the abundance of *Lactobacillus* and *Bifidobacterium*[119]. Resveratrol attenuates trimethylamine-N-oxide-induced atherosclerosis by remodeling the gut microbiota and increasing the relative abundance of *Bacteroides*, *Lactobacillus*, *Bifidobacterium*, and *Akkermansia* in mice [120].

### Polyamines

Polyamines are organic compounds containing more than two amino groups such as putrescine, spermidine, and spermine [121]. Polyamines are natural compounds found in various foods that play a role in many physiological processes. While they are generally safe when consumed in moderation through the diet, normal supplementation has not been reported potential side effects [122].

Unlike the compounds that have appeared so far, polyamines are originally present in the cells of all organisms. Polyamines in vivo are synthesized in their own cells, as well as those produced by gut bacteria and derived from dietary sources, which are absorbed and utilized. Polyamines are involved in many cellular processes, including DNA maintenance, RNA processing, translation, and protein activation [123]. Spermidine (Fig. 4) is a well-studied polyamine present in many fermented foods such as yogurt and miso. Spermidine administration extended the lifespan of mice and improved cardiac dysfunction and metabolic syndrome by inducing autophagy [124, 125]. Polyamine production promoted by gut bacterial has been shown to prolong lifespan in mice [126].

Administration of a symbiotic comprising arginine—a precursor of polyamines in microbial metabolism—and a certain beneficial bacterium of *Bifidobacterium animalis* subsp*. lactis* LKM512 strain upregulates putrescine in the colon and increases spermidine in the blood [127]. A symbiotic is defined as “a mixture comprising live microorganisms and substrates selectively utilized by host microorganisms that confer a benefit on the host” [128]. In another preclinical study, spermidine altered the composition of the gut microbiota in obese mice specifically by increasing the abundance of the organic acid-producing bacteria Lachnospiraceae [129].

## Discussion and conclusion

A “healthy intestinal environment” means having a gut that has a good balance of helpful microorganisms and avoids harmful ones [130, 131]. This can be noticed in several ways, such as regular bowel movements, absence of gastrointestinal symptoms, no chronic inflammation, strong immune system, and normal nutrient absorption. Basically, it means having a gut that works well and keeps you healthy. In this study, we reported that CRMs may extend lifespan partly through the gut microbiota, as we found that all CRM alter the gut microbiota (Table 2). Furthermore, we discovered that CRMs do not necessarily increase the diversity of the gut microbes. CRMs increase the abundance of one or more specific beneficial species, such as *Akkermansia*, *Bifidobacterium*, *Lactobacillus*, *and Bacteroides*. CRMs seem to alter the microbiota favorably, especially with respect to its anti-diabetic and anti-obese effects. Additionally, some CRMs also reduce the number of harmful species. Conventionally, beneficial substances that promote intestinal health are known as prebiotics and are defined as “substrates that are selectively utilized by host microorganisms conferring a health benefit.” An example of a prebiotic is fructo-oligosaccharides [132], although probiotics that are live microorganisms confer a health benefit on the host [133]. However, prebiotics non-specifically stimulate the growth of many members of the intestinal microbiomes that are both beneficial and harmful to human health. Recently, next-generation prebiotics have been proposed to selectively promote the growth of beneficial bacteria, in contrast to conventional prebiotics [134]. In this regard, CRMs act as the next-generation prebiotics. In addition, some preclinical studies have reported that the microbial diversity or weight of the cecum did not increase due to CRMs. This is also contrary to the action of conventional prebiotics, which increase the microbial diversity or weight of the cecum. Taken together, the effect of CRM on the gut is different from that of conventional prebiotics but seems similar to that of next-generation prebiotics.

**Table 2:** Influence of CRMs on intestinal microbiome

CRMs	Subject	Diet condition	Diversity^1^	Bacteria on increase	Bacteria on decrease	References
Metformin	Mice	High-fat diet	NR	*Akkermansia*	NR	[36]
	Mice	Normal diet	NR	NR	*Bacteroides fragilis*	[37]
	Human	Normal diet	NR	*Escherichia/Shigella*, *Bilophila wadsworthia*	*Intestinibacter*,* Clostridium*	[39]
Acarbose	Mice	Normal diet	NR	Muribaculaceae	Lactobacillaceae, Erysipelotrichaceae	[48]
	Human	Normal diet	Not changed^2^	Lactobacillaceae, Ruminococcaceae, Veillonellaceae	Ruminococcaceae, Lachnospiraceae	[49]
SGLT2 inhibitor	Mice	Normal diet	NR	*Bacteroides*, *Odoribacter*	*Oscillibacter*	[53]
	Mice	Normal diet	NR	*Bifidobacterium*	*Oscillospira*	[55]
	Human	Normal diet	NR	*Roseburia*, *Eubacterium*, *Faecalibacterium*	*Escherichia-Shigella*, *Bilophila*, *Hungatella*	[56]
D-Glucosamine	C57BL6 mice	High-fat diet	Not changed^3^	*Bifidobacterium*, *Akkermansia*, *Lactobacillus*, *Allobaculum*	*Roseburia*, *Desulfovibrio*, *Oscillibacter*, *Intestinimonas*	[61]
	Human	Normal diet	Not changed^2^	NR	Peptococcaceae, Bacillaceae	[66]
D-Allulose	C57BL6 mice	High-fat diet	Increase^2^	*Coprococcus*	NR	[70]
	C57BL7 mice	High-fat diet	NR	*Lactobacillus*, *Coprococcus*, *Coprobacillus*	*Turicibacter*, Clostridiaceae, *Dorea*, Erysipelotrichaceae	[72]
	Human	Normal diet	NR	*Coprococcus*, *Blautia*	NR	[73]
D-Allose	C57BL6 mice	Normal diet	NR	*Bacteroides acidifaciens*, *Akkermansia muciniphila*	*Blautia*, Lachnospiraceae	[82]
Rapamycin	Mice	Normal diet	NR	*Marinilabiliaceae*, *Turicibacter*	NR	[86]
Resveratrol	Mice	Normal diet	NR	*Lactobacillus*,* Bifidobacterium*	*Enterococcus faecalis*	[92]
	Mice	Normal diet	NR	*Bacteroides*, *Lactobacillus*, *Bifidobacterium*, *Akkermansia*	NR	[93]
Polyamines	Mice	Normal diet	NR	Lachnospiraceae	NR	[100]

^1^There are two main types of diversity that are commonly studied in gut microbiome: α-diversity and β-diversity. ^2^α-diversity refers to the diversity within a single sample. ^3^β-diversity refers to the differences in microbial community composition between different samples. NR, not reported

Two important papers demonstrating the association between gut microbiota and lifespan have been recently reported. One research group found that certain microbial taxa, including *Prevotella*, were associated with a longer lifespan in a Finnish population cohort [135]. These results suggest that the gut microbiota may play a role in promoting healthy aging and longevity. Interestingly, they also found that higher levels of SCFAs in fecal samples were associated with a longer lifespan, which suggests that gut microbial metabolism may be an important factor in promoting healthy aging. However, the effect of CRM drugs on SCFA production has not been reported. The other research group found that gut microbiota diversity was associated with biological age, as measured by the epigenetic clock, in a Dutch population cohort [136]. Specifically, individuals with a more diverse gut microbiota had a younger biological age. They also identified certain microbial taxa, such as Faecalibacterium, that were associated with a younger biological age. These results suggest that the gut microbiota may play a role in regulating the aging process. CRM drugs, such as next-generation prebiotics, may approach the gut microbiota of younger biological age in that there are changes in specific bacterial communities.

CRM drugs can extend the lives of healthy individuals. Notably, D-glucosamine has shown low mortality in humans in multiple large epidemiological studies [137, 138]. However, the underlying detailed mechanisms remain unclear. In particular, the exact mechanism underlying the life-prolonging effects of these CRMs needs to be elucidated both indirectly from a microbiome perspective and directly through targets in the host. Note that we have important limitations of the many studies cited in this review, although we concluded that CRMs influence gut microbes. At least four limitations should be considered. First of all, it has not been still obvious to a borderline of eliciting a significant phenotypic change in health status. For instance, it is too difficult to consider this change as significant, if a bacterium that is the 0.01% abundant increases into 0.5% (50-fold) by an intervention, yet remains at the bottom of prevalence in the host (for instance, the criteria 1%). We should keep in mind that the significance of changes in microbiota composition might depend on many factors, including the specific bacterial taxa involved, the individual host, and the overall microbial community structure. Next, in many reports cited in this review, studies may not adequately control for lifestyle factors that can influence the gut microbiota, such as diet, exercise, stress, and medication use, among others. Thus, it is important to acknowledge that not all studies are of equal quality, and some may have limitations that affect the robustness of their conclusions. As third limitation, in many reports, animal experiments using antibiotics were not conducted. The effect of altered microbe by CRMs on lifespan has not been elucidated except acarbose. Ideally, the effect on lifespan must be examined concurrently in combination with antibiotics to cancel the influence of intestinal bacteria on CRM. Lastly, as fourth limitation, it is a matter of species difference of the many studies cited in this review. Mice, rats, and human populations are very different in composition (diversity and relative abundance) [139]. Due to these differences between humans and mice, much caution is required when interpreting the results of studies in mice [140]. Also, the differences among human subjects entail caution when interpreting clinical studies. This is because human intestinal microflora is traditionally classified into three types: *Bacteroides*, *Prevotella*, and *Ruminococcus* type [141]. After that, other study showed the four types by dividing the former *Bacteroides* type [142]. Therefore, clinical trials should be designed based on these some types of microflora. Clinical trials related to CRM are expected to be long-term trials; therefore, sufficient information must be gathered regarding the participants in advance. Thus, prior studies in humans using an intestinal model independent of diet condition might be necessary to ensure the appropriateness of conducting clinical trials from an ethical or economic point of view [143, 144], because dietary conditions of the participant significantly influence the results of clinical trials. Confirming that the effects of CRM drugs in humans on the intestinal microbiome and related biomarkers mimic those of CRs is necessary. Further research on lifespan extension via gut microbiome modulation should be conducted in order to help achieve an anti-senescence goal.

## Abbreviations

AMPK: AMP-activated protein kinase
CR: Calorie restriction
CRM: CR mimetic

## References

[1] Madeo F, Pietrocola F, Eisenberg T, Kroemer G. Caloric restriction mimetics: towards a molecular definition. Nat Rev Drug Discov [Internet]. Nature Publishing Group; 2014;13:727–40. 10.1038/nrd4391. 10.1038/nrd4391 25212602

[2] Shintani H, Shintani T, Ashida H, Sato M (2018). Calorie restriction mimetics: upstream-type compounds for modulating glucose metabolism. Nutrients.

[3] Most J, Tosti V, Redman LM, Fontana L (2017). Calorie restriction in humans: an update. Ageing Res Rev.

[4] Shintani T (2020). Human antiaging research: a viewpoint from food science on calorie restriction mimetics. Food Res.

[5] Martin CK, Bhapkar M, Pittas AG, Pieper CF, Das SK, Williamson DA (2016). Effect of calorie restriction on mood, quality of life, sleep, and sexual function in healthy nonobese adults. JAMA Intern Med.

[6] Dorling JL, van Vliet S, Huffman KM, Kraus WE, Bhapkar M, Pieper CF (2021). Effects of caloric restriction on human physiological, psychological, and behavioral outcomes: highlights from CALERIE phase 2. Nutr Rev.

[7] Ingram DK, Anson RM, de Cabo R, Mamczarz J, Zhu M, Mattison J (2004). Development of calorie restriction mimetics as aprolongevity strategy. Ann N Y Acad Sci..

[8] Madeo F, Pietrocola F, Eisenberg T, Kroemer G (2014). Caloric restriction mimetics: towards a molecular definition. Nat Rev Drug Discov.

[9] Madeo F, Carmona-Gutierrez D, Hofer SJ, Kroemer G (2019). Caloric restriction mimetics against age-associated disease: targets, mechanisms, and therapeutic potential. Cell Metab.

[10] Mark LA, Donald IK, George RS (1998). 2-Deoxy-D-glucose feeding in rats mimics physiologic effects of calorie restriction. J Anti Aging Med.

[11] Ingram DK, Roth GS (2011). Glycolytic inhibition as a strategy for developing calorie restriction mimetics. Exp Gerontol..

[12] Ingram DK, Roth GS (2015). Calorie restriction mimetics: can you have your cake and eat it, too?. Ageing Res Rev..

[13] Ingram DK, Roth GS (2020). Glycolytic inhibition: an effective strategy for developing calorie restriction mimetics.

[14] Lynch SV, Pedersen O (2016). The human intestinal microbiome in health and disease. New England Journal of Medicine..

[15] Ottman N, Smidt H, de Vos WM, Belzer C (2012). Thefunction of our microbiota: who is out there and what do they do?. Front Cell Infect Microbiol..

[16] Sekirov I, Russell SL, Antunes LCM, Finlay BB (2010). Gut Microbiota in Health and Disease. Physiol Rev.

[17] Lynch S v., Pedersen O. The human intestinal microbiome in health and disease. New England J Med 2016;375:2369–79. 10.1056/NEJMra1600266 27974040

[18] Odamaki T, Kato K, Sugahara H, Hashikura N, Takahashi S, Xiao J (2016). Age-related changes in gut microbiota composition from newborn to centenarian: a cross-sectional study. BMC Microbiol..

[19] Ríos-Covián D, Ruas-Madiedo P, Margolles A, Gueimonde M, de los Reyes-Gavilán CG, Salazar N. Intestinal short chain fatty acids and their link with diet and human health. Front Microbiol. 2016;7:185. 10.3389/fmicb.2016.00185 PMC4756104 26925050

[20] Koh A, De Vadder F, Kovatcheva-Datchary P, Bäckhed F (2016). From dietary fiber to host physiology: short-chain fatty acids as key bacterial metabolites. Cell.

[21] Holscher HD (2017). Dietary fiber and prebiotics and the gastrointestinal microbiota. Gut Microbes.

[22] Hamer HM, Jonkers D, Venema K, Vanhoutvin S, Troost FJ, Brummer R-J (2007). Review article: the role of butyrate on colonic function. Aliment Pharmacol Ther.

[23] George Kerry R, Patra JK, Gouda S, Park Y, Shin H-S, Das G (2018). Benefaction of probiotics for human health: a review. J Food Drug Anal.

[24] Singh RK, Chang H-W, Yan D, Lee KM, Ucmak D, Wong K (2017). Influence of diet on the gut microbiome and implications for human health. J Transl Med.

[25] Hartstra AV, Bouter KEC, Bäckhed F, Nieuwdorp M (2015). Insights into the role of the microbiome in obesity and type 2 diabetes. Diabetes Care.

[26] David LA, Maurice CF, Carmody RN, Gootenberg DB, Button JE, Wolfe BE (2014). Diet rapidly and reproducibly alters the human gut microbiome. Nature.

[27] Yang J-Y, Lee Y-S, Kim Y, Lee S-H, Ryu S, Fukuda S (2017). Gut commensal Bacteroides acidifaciens prevents obesity and improves insulin sensitivity in mice. Mucosal Immunol.

[28] Turnbaugh PJ, Ley RE, Mahowald MA, Magrini V, Mardis ER, Gordon JI (2006). An obesity-associated gut microbiome with increased capacity for energy harvest. Nature.

[29] Markowiak-Kopeć P, Śliżewska K (2020). The effect of probiotics on the production of short-chain fatty acids by human intestinal microbiome. Nutrients.

[30] Ley RE, Bäckhed F, Turnbaugh P, Lozupone CA, Knight RD, Gordon JI (2005). Obesity alters gut microbial ecology. Proc Natl Acad Sci.

[31] Mariat D, Firmesse O, Levenez F, Guimarăes V, Sokol H, Doré J (2009). The Firmicutes/Bacteroidetes ratio of the human microbiota changes with age. BMC Microbiol.

[32] Wu C-S, Muthyala SDV, Klemashevich C, Ufondu AU, Menon R, Chen Z (2021). Age-dependent remodeling of gut microbiome and host serum metabolome in mice. Aging.

[33] Biagi E, Franceschi C, Rampelli S, Severgnini M, Ostan R, Turroni S (2016). Gut microbiota and extreme longevity. Curr Biol.

[34] Ang QY, Alexander M, Newman JC, Tian Y, Cai J, Upadhyay V (2020). Ketogenic diets alter the gut microbiome resulting in decreased intestinal Th17 cells. Cell.

[35] Olson CA, Vuong HE, Yano JM, Liang QY, Nusbaum DJ, Hsiao EY (2018). The gut microbiota mediates the anti-seizure effects of the ketogenic diet. Cell.

[36] Russo M, Fabersani E, Abeijón-Mukdsi M, Ross R, Fontana C, Benítez-Páez A (2016). Lactobacillus fermentum CRL1446 ameliorates oxidative and metabolic parameters by increasing intestinal feruloyl esterase activity and modulating microbiota in caloric-restricted mice. Nutrients.

[37] Ruiz A, Cerdó T, Jáuregui R, Pieper DH, Marcos A, Clemente A (2017). One-year calorie restriction impacts gut microbial composition but not its metabolic performance in obese adolescents. Environ Microbiol.

[38] Zhang Y, Qi H, Wang L, Hu C, Gao A, Wu Q, et al. Fasting and refeeding triggers specific changes in bile acid profiles and gut microbiota. J Diabetes. 2023;15:165–80 10.1111/1753-0407.13356 PMC9934961 36682739

[39] Chaudhury A, Duvoor C, Reddy Dendi VS, Kraleti S, Chada A, Ravilla R (2017). Clinical review of antidiabetic drugs: implicationsfor type 2 diabetes mellitus management. Front Endocrinol (Lausanne)..

[40] Bouchoucha M, Uzzan B, Cohen R (2011). Metformin and digestive disorders. Diabetes Metab.

[41] Rojas LBA, Gomes MB (2013). Metformin: an old but still the best treatment for type 2 diabetes. Diabetol Metab Syndr.

[42] Bailey CJ, Turner RC (1996). Metformin. N Engl J Med.

[43] Shurrab NT, Arafa E-SA. Metformin: a review of its therapeutic efficacy and adverse effects. Obes Med. 2020;17:100186.

[44] Shintani H, Shintani T (2020). Effects of antidiabetic drugs that cause glucose excretion directly from the body on mortality. Med Drug Discov.

[45] Zhou G, Myers R, Li Y, Chen Y, Shen X, Fenyk-Melody J (2001). Role of AMP-activated protein kinase in mechanism of metformin action. J Clin Investig.

[46] Foretz M, Guigas B, Bertrand L, Pollak M, Viollet B (2014). Metformin: from mechanisms of action to therapies. Cell Metab.

[47] Morita Y, Nogami M, Sakaguchi K, Okada Y, Hirota Y, Sugawara K (2020). Enhanced release of glucose into the intraluminal space of the intestine associated with metformin treatment as revealed by [18F]fluorodeoxyglucose PET-MRI. Diabetes Care.

[48] Pascale A, Marchesi N, Govoni S, Coppola A, Gazzaruso C (2019). The role of gut microbiota in obesity, diabetes mellitus, and effect of metformin: new insights into old diseases. Curr Opin Pharmacol.

[49] Shin N-R, Lee J-C, Lee H-Y, Kim M-S, Whon TW, Lee M-S (2014). An increase in the *Akkermansia* spp. population induced by metformin treatment improves glucose homeostasis in diet-induced obese mice. Gut..

[50] Sun L, Xie C, Wang G, Wu Y, Wu Q, Wang X (2018). Gut microbiota and intestinal FXR mediate the clinical benefits of metformin. Nat Med.

[51] Wu H, Esteve E, Tremaroli V, Khan MT, Caesar R, Mannerås-Holm L (2017). Metformin alters the gut microbiome of individuals with treatment-naive type 2 diabetes, contributing to the therapeutic effects of the drug. Nat Med.

[52] Bryrup T, Thomsen CW, Kern T, Allin KH, Brandslund I, Jørgensen NR (2019). Metformin-induced changes of the gut microbiota in healthy young men: results of a non-blinded, one-armed intervention study. Diabetologia.

[53] Forslund K, Hildebrand F, Nielsen T, Falony G, le Chatelier E, Sunagawa S (2015). Disentangling type 2 diabetes and metformin treatment signatures in the human gut microbiota. Nature.

[54] Chiasson J-L, Josse RG, Gomis R, Hanefeld M, Karasik A, Laakso M (2002). Acarbose for prevention of type 2 diabetes mellitus: the STOP-NIDDM randomised trial. Lancet.

[55] Hollander P (1992). Safety profile of acarbose, an α-glucosidase inhibitor. Drugs.

[56] Yee HS, Fong NT (1996). A review of the safety and efficacy of acarbose in diabetes mellitus. Pharmacotherapy.

[57] Lebovitz HE (1997). Alpha-glucosidase inhibitors. Endocrinol Metab Clin North Am.

[58] Campbell LK, White JR, Campbell RK (1996). Acarbose: its role in the treatment of diabetes mellitus. Ann Pharmacother.

[59] Harrison DE, Strong R, Allison DB, Ames BN, Astle CM, Atamna H (2014). Acarbose, 17-α-estradiol, and nordihydroguaiaretic acid extend mouse lifespan preferentially in males. Aging Cell.

[60] Dehghan-Kooshkghazi M, Mathers JC (2004). Starch digestion, large-bowel fermentation and intestinal mucosal cell proliferation in rats treated with the α-glucosidase inhibitor acarbose. Br J Nutr.

[61] Weaver GA, Tangel CT, Krause JA, Parfitt MM, Jenkins PL, Rader JM (1997). Acarbose enhances human colonic butyrate production. J Nutr.

[62] Weaver GA, Tangel CT, Krause JA, Parfitt MM, Stragand JJ, Jenkins PL (2000). Biomarkers of human colonic cell growth are influenced differently by a history of colonic neoplasia and the consumption of acarbose. J Nutr.

[63] Nagata N, Nishijima S, Miyoshi-Akiyama T, Kojima Y, Kimura M, Aoki R (2022). Population-level metagenomics uncovers distinct effects of multiple medications on the human gut microbiome. Gastroenterology.

[64] Smith BJ, Miller RA, Ericsson AC, Harrison DC, Strong R, Schmidt TM (2019). Changes in the gut microbiome and fermentation products concurrent with enhanced longevity in acarbose-treated mice. BMC Microbiol.

[65] Zhang X, Fang Z, Zhang C, Xia H, Jie Z, Han X (2017). Effects of acarbose on the gut microbiota of prediabetic patients: a randomized, double-blind, controlled crossover trial. Diabetes Therapy.

[66] Scheen AJ (2019). An update on the safety of SGLT2 inhibitors. Expert Opin Drug Saf.

[67] McGill JB, Subramanian S (2019). Safety of sodium-glucose co-transporter 2 inhibitors. Am J Med..

[68] Kalra S (2014). Sodium Glucose Co-Transporter-2 (SGLT2) Inhibitors: a review of their basic and clinical pharmacology. Diabetes Ther.

[69] Lee PC, Ganguly S, Goh S-Y (2018). Weight loss associated with sodium-glucose cotransporter-2 inhibition: a review of evidence and underlying mechanisms. Obes Rev.

[70] Ho H, Kikuchi K, Oikawa D, Watanabe S, Kanemitsu Y, Saigusa D, et al. SGLT‐1‐specific inhibition ameliorates renal failure and alters the gut microbial community in mice with adenine‐induced renal failure. Physiol Rep. 2021;9:15092. 10.14814/phy2.15092 PMC8683788 34921520

[71] Deng L, Yang Y, Xu G. Empagliflozin ameliorates type 2 diabetes mellitus-related diabetic nephropathy via altering the gut microbiota. Biochim Biophys Acta (BBA) - Molec Cell Biol Lipids. 2022;1867:159234. 10.1016/j.bbalip.2022.159234 36185030

[72] Mishima E, Fukuda S, Kanemitsu Y, Saigusa D, Mukawa C, Asaji K (2018). Canagliflozin reduces plasma uremic toxins and alters the intestinal microbiota composition in a chronic kidney disease mouse model. Am J Physiol-Renal Physiol.

[73] Konikoff T, Gophna U (2016). Oscillospira : a central, enigmatic component of the human gut microbiota. Trends Microbiol.

[74] Deng X, Zhang C, Wang P, Wei W, Shi X, Wang P (2022). Cardiovascular benefits of empagliflozin are associated with gut microbiota and plasma metabolites in type 2 diabetes. J Clin Endocrinol Metab.

[75] Anderson JW, Nicolosi RJ, Borzelleca JF (2005). Glucosamine effects in humans: a review of effects on glucose metabolism, side effects, safety considerations and efficacy. Food Chem Toxicol.

[76] Hathcock JN, Shao A (2007). Risk assessment for glucosamine and chondroitin sulfate. Regul Toxicol Pharmacol.

[77] Dalirfardouei R, Karimi G, Jamialahmadi K (2016). Molecular mechanisms and biomedical applications of glucosamine as a potential multifunctional therapeutic agent. Life Sci.

[78] Shintani T, Yamazaki F, Katoh T, Umekawa M, Matahira Y, Hori S (2010). Glucosamine induces autophagy via anmTOR-independent pathway. Biochem Biophys Res Commun..

[79] Shintani T, Kosuge Y, Ashida H (1999). Glucosamine extends the lifespan of caenorhabditis elegans via autophagy induction glucosamine extends nematode lifespan via autophagy induction. J Appl Glycosci.

[80] Shintani H, Ashida H, Shintani T (2021). Shifting the focus of D-glucosamine from a dietary supplement for knee osteoarthritis to a potential anti-aging drug. Human Nutr Metab.

[81] Yoon SY, Narayan VP (2022). Genetically predicted glucosamine and longevity: a Mendelian randomization study. Clin Nutr ESPEN.

[82] Setnikar I, Rovati L (2011). Absorption, distribution, metabolism and excretion of glucosamine sulfate. Arzneimittelforschung.

[83] Yuan X, Zheng J, Ren L, Jiao S, Feng C, Du Y, et al. Glucosamine ameliorates symptoms of high-fat diet-fed mice by reversing imbalanced gut microbiota. Front Pharmacol. 2021;12:694107. 10.3389/fphar.2021.694107 PMC8209492 34149435

[84] Tamanai-Shacoori Z, Smida I, Bousarghin L, Loreal O, Meuric V, Fong SB (2017). *Roseburia* spp.: a marker of health?. Future Microbiol..

[85] Finegold SM (2011). Desulfovibrio species are potentially important in regressive autism. Med Hypotheses.

[86] Murros KE, Huynh VA, Takala TM, Saris PEJ. Desulfovibrio bacteria are associated with Parkinson’s disease. Front Cell Infect Microbiol. 2021;11:652617. 10.3389/fcimb.2021.652617 PMC8126658 34012926

[87] Berry D, Reinisch W (2013). Intestinal microbiota: a source of novel biomarkers in inflammatory bowel diseases?. Best Pract Res Clin Gastroenterol.

[88] Moon JM, Finnegan P, Stecker RA, Lee H, Ratliff KM, Jäger R (2021). Impact of glucosamine supplementation on gut health. Nutrients.

[89] Hossain A, Yamaguchi F, Matsuo T, Tsukamoto I, Toyoda Y, Ogawa M (2015). Rare sugar D-allulose: potential role and therapeutic monitoring in maintaining obesity and type 2 diabetes mellitus. Pharmacol Ther.

[90] Han Y, Choi BR, Kim SY, Kim S-B, Kim YH, Kwon E-Y, et al. Gastrointestinal tolerance of D-allulose in healthy and young adults. A non-randomized controlled trial. Nutrients. 2018;10:2010. 10.3390/nu10122010 PMC6315886 30572580

[91] Shintani T, Yamada T, Hayashi N, Iida T, Nagata Y, Ozaki N (2017). Rare sugar syrup containing D-allulose but not high-fructosecorn syrup maintains glucose tolerance and insulin sensitivity partly viahepatic glucokinase translocation in wistar rats. J Agric Food Chem..

[92] Shintani T, Sakoguchi H, Yoshihara A, Izumori K, Sato M (2017). D -Allulose, a stereoisomer of D -fructose, extends Caenorhabditis elegans lifespan through a dietary restriction mechanism: a new candidate dietary restriction mimetic. Biochem Biophys Res Commun..

[93] Iida T, Hayashi N, Yamada T, Yoshikawa Y, Miyazato S, Kishimoto Y, et al. Failure of d-psicose absorbed in the small intestine to metabolize into energy and its low large intestinal fermentability in humans. Metabolism. 2010;59:206–14. 10.1016/j.metabol.2009.07.018 19765780

[94] Han Y, Park H, Choi B-R, Ji Y, Kwon E-Y, Choi M-S (2020). Alteration of microbiome profile by D-allulose in amelioration of high-fat-diet-induced obesity in mice. Nutrients.

[95] Liu P, Wang Y, Yang G, Zhang Q, Meng L, Xin Y (2021). The role of short-chain fatty acids in intestinal barrier function, inflammation, oxidative stress, and colonic carcinogenesis. Pharmacol Res.

[96] Han Y, Yoon J, Choi M (2020). Tracing the anti-inflammatory mechanism/triggers of D-allulose: a profile study of microbiome composition and mRNA expression in diet-induced obese mice. Mol Nutr Food Res.

[97] Shimonaka A, Yamaji T, Dobashi H, Kitamura N, Iida T. Composition for promoting proliferation of genus coprococcus bacterium [Internet]. Japan; 2018 [cited 2022 Nov 6]. https://patents.google.com/patent/JP2020074695A/en.

[98] Liu X, Mao B, Gu J, Wu J, Cui S, Wang G, et al. *Blautia* —a new functional genus with potential probiotic properties? Gut Microbes. 2021;13:1–21. 10.1080/19490976.2021.1875796 PMC7872077 33525961

[99] Shintani H, Shintani T, Sato M, Sato. D-Allose M. D-Allose, a trace component in human serum, and its pharmaceutical applicability Citation. Its Pharmaceutical Applicability. Int J Appl Biol Pharm Technol. 2020;11:200–13.

[100] Lim Y-R, Oh D-K (2011). Microbial metabolism and biotechnological production of d-allose. Appl Microbiol Biotechnol..

[101] Chen Z, Chen J, Zhang W, Zhang T, Guang C, Mu W (2018). Recent research on the physiological functions, applications, andbiotechnological production of d-allose. Appl Microbiol Biotechnol..

[102] Tomoya S, Kazuhiro O, Hirofumi S, Masashi S (2013). Rare sugars D-psicose and D-allose as calorie restriction mimetic-anti-metabolic syndrome effects and anti-aging effects. J Brewing Soc Jpn.

[103] Shintani T, Sakoguchi H, Yoshihara A, Izumori K, Sato M (2019). D-Allose, a stereoisomer of d-glucose, extends the lifespan of Caenorhabditis elegans via sirtuin and insulin signaling. J Appl Glycosci (1999).

[104] Iga Y, Matsuo T (2010). D-Allose metabolism in rats. Nippon Eiyo Shokuryo Gakkaishi.

[105] Kitagawa M, Tanaka M, Yoshikawa Y, Iida T, Kishimoto Y (2018). Evaluation of ABSORPTION and fermentability of D-mannose, D-sorbose, and D-allose in humans. Luminacoids Res.

[106] Shintani T, Yanai S, Kanasaki A, Tanaka M, Iida T, Ozawa G, et al. Long-term D-allose administration favorably alters the intestinal environment in aged male mice. J Appl Glycosci (1999) [Internet]. 2022;jag.JAG-2022_0005. https://www.jstage.jst.go.jp/article/jag/advpub/0/advpub_jag.JAG-2022_0005/_article. 10.5458/jag.jag.JAG-2022_0005 PMC9720632 36531693

[107] Guba M, von Breitenbuch P, Steinbauer M, Koehl G, Flegel S, Hornung M (2002). Rapamycin inhibits primary and metastatic tumor growth by antiangiogenesis: involvement of vascular endothelial growth factor. Nat Med.

[108] McCormack FX, Inoue Y, Moss J, Singer LG, Strange C, Nakata K (2011). Efficacy and safety of sirolimus in lymphangioleiomyomatosis. N Engl J Med.

[109] Lamming DW, Ye L, Sabatini DM, Baur JA (2013). Rapalogs and mTOR inhibitors as anti-aging therapeutics. J Clin Investig.

[110] Harrison DE, Strong R, Sharp ZD, Nelson JF, Astle CM, Flurkey K (2009). Rapamycin fed late in life extends lifespan in genetically heterogeneous mice. Nature.

[111] Bitto A, Ito TK, Pineda V v, LeTexier NJ, Huang HZ, Sutlief E, et al. Transient rapamycin treatment can increase lifespan and healthspan in middle-aged mice. Elife. 2016;5:e16351. 10.7554/eLife.16351 PMC4996648 27549339

[112] Jung M-J, Lee J, Shin N-R, Kim M-S, Hyun D-W, Yun J-H (2016). Chronic repression of mTOR complex 2 Induces changes in the gut microbiota of diet-induced obese mice. Sci Rep.

[113] Siemann EH, Creasy LL (1992). Concentration of the phytoalexin resveratrol in wine. Am J Enol Vitic..

[114] Shaito A, Posadino AM, Younes N, Hasan H, Halabi S, Alhababi D, et al. Potential adverse effects of resveratrol: a literature review. Int J Mol Sci. 2020;21:2084. 10.3390/ijms21062084 PMC7139620 32197410

[115] de Ligt M, Timmers S, Schrauwen P. Resveratrol and obesity: Can resveratrol relieve metabolic disturbances? Biochim Biophys Acta (BBA) - Molec Basis Dis 2015;1852:1137–44. 10.1016/j.bbadis.2014.11.012 25446988

[116] Pan Y, Zhang H, Zheng Y, Zhou J, Yuan J, Yu Y (2017). resveratrol exerts antioxidant effects by activating SIRT2 to deacetylate Prx1. Biochemistry.

[117] Baur JA, Pearson KJ, Price NL, Jamieson HA, Lerin C, Kalra A (2006). Resveratrol improves health and survival of mice on a high-calorie diet. Nature.

[118] da Luz PL, Tanaka L, Brum PC, Dourado PMM, Favarato D, Krieger JE (2012). Red wine and equivalent oral pharmacological doses of resveratrol delay vascular aging but do not extend life span in rats. Atherosclerosis.

[119] Wang P, Li D, Ke W, Liang D, Hu X, Chen F (2020). Resveratrol-induced gut microbiota reduces obesity in high-fat diet-fed mice. Int J Obes.

[120] Chen M, Yi L, Zhang Y, Zhou X, Ran L, Yang J, et al. Resveratrol attenuates trimethylamine- *N* -oxide (TMAO)-induced atherosclerosis by regulating TMAO synthesis and bile acid metabolism via remodeling of the gut microbiota. mBio. 2016;7:e02210-15. 10.1128/mBio.02210-15 PMC4817264 27048804

[121] Pegg AE, McCann PP. Polyamine metabolism and function. Am J Physiol-Cell Physiol. 1982;243:C212–21. 10.1152/ajpcell.1982.243.5.C212 6814260

[122] Schwarz C, Stekovic S, Wirth M, Benson G, Royer P, Sigrist SJ (2018). Safety and tolerability of spermidine supplementation in mice and older adults with subjective cognitive decline. Aging.

[123] Childs AC, Mehta DJ, Gerner EW (2003). Polyamine-dependent gene expression. Cell Mol Life Sci.

[124] Eisenberg T, Abdellatif M, Schroeder S, Primessnig U, Stekovic S, Pendl T (2016). Cardioprotection and lifespan extension by the natural polyamine spermidine. Nat Med.

[125] Eisenberg T, Knauer H, Schauer A, Büttner S, Ruckenstuhl C, Carmona-Gutierrez D (2009). Induction of autophagy by spermidine promotes longevity. Nat Cell Biol.

[126] Matsumoto M, Kurihara S, Kibe R, Ashida H, Benno Y (2011). Longevity in mice is promoted by probiotic-induced suppression of colonic senescence dependent on upregulation of gut bacterial polyamine production. PLoS ONE.

[127] Kibe R, Kurihara S, Sakai Y, Suzuki H, Ooga T, Sawaki E (2015). Upregulation of colonic luminal polyamines produced by intestinal microbiota delays senescence in mice. Sci Rep.

[128] Swanson KS, Gibson GR, Hutkins R, Reimer RA, Reid G, Verbeke K (2020). The International Scientific Association for Probiotics and Prebiotics (ISAPP) consensus statement on the definition and scope of synbiotics. Nat Rev Gastroenterol Hepatol.

[129] Ma L, Ni Y, Wang Z, Tu W, Ni L, Zhuge F (2020). Spermidine improves gut barrier integrity and gut microbiota function in diet-induced obese mice. Gut Microbes.

[130] Rinninella E, Raoul P, Cintoni M, Franceschi F, Miggiano G, Gasbarrini A (2019). What is the healthy gut microbiota composition? A changing ecosystem across age, environment, diet, and diseases. Microorganisms.

[131] Round JL, Mazmanian SK (2009). The gut microbiota shapes intestinal immune responses during health and disease. Nat Rev Immunol.

[132] Gibson GR, Hutkins R, Sanders ME, Prescott SL, Reimer RA, Salminen SJ (2017). Expert consensus document: the International Scientific Association for Probiotics and Prebiotics (ISAPP) consensus statement on the definition and scope of prebiotics. Nat Rev Gastroenterol Hepatol.

[133] Roberfroid M, Gibson GR, Hoyles L, McCartney AL, Rastall R, Rowland I (2010). Prebiotic effects: metabolic and health benefits. Br J Nutr.

[134] Lordan C, Thapa D, Ross RP, Cotter PD (2020). Potential for enriching next-generation health-promoting gut bacteria through prebiotics and other dietary components. Gut Microbes.

[135] Salosensaari A, Laitinen V, Havulinna AS, Meric G, Cheng S, Perola M (2021). Taxonomic signatures of cause-specific mortality risk in human gut microbiome. Nat Commun.

[136] Wilmanski T, Diener C, Rappaport N, Patwardhan S, Wiedrick J, Lapidus J (2021). Gut microbiome pattern reflects healthy ageing and predicts survival in humans. Nat Metab.

[137] Li Z-H, Gao X, Chung VC, Zhong W-F, Fu Q, Lv Y-B (2020). Associations of regular glucosamine use with all-cause and cause-specific mortality: a large prospective cohort study. Ann Rheum Dis.

[138] Pocobelli G, Kristal AR, Patterson RE, Potter JD, Lampe JW, Kolar A (2010). Total mortality risk in relation to use of less-common dietary supplements. Am J Clin Nutr..

[139] Kobayashi R, Nagaoka K, Nishimura N, Koike S, Takahashi E, Niimi K, et al. Comparison of the fecal microbiota of two monogastric herbivorous and five omnivorous mammals. Animal Sci J. 2020;91:e13366. 10.1111/asj.13366 PMC7216987 32285557

[140] Nguyen TLA, Vieira-Silva S, Liston A, Raes J (2015). How informative is the mouse for human gut microbiota research?. Dis Model Mech.

[141] Wu GD, Chen J, Hoffmann C, Bittinger K, Chen Y-Y, Keilbaugh SA (1979). Linking long-term dietary patterns with gut microbial enterotypes. Science.

[142] Vandeputte D, Kathagen G, D’hoe K, Vieira-Silva S, Valles-Colomer M, Sabino J, et al. Quantitative microbiome profiling links gut community variation to microbial load. Nature. 2017;551:507–11. 10.1038/nature24460 29143816

[143] Hoshi N, Inoue J, Sasaki D, Sasaki K (2021). The Kobe University Human Intestinal Microbiota Model for gut intervention studies. Appl Microbiol Biotechnol.

[144] Li C, Zhang X (2022). Current in vitro and animal models for understanding foods: human gut–microbiota interactions. J Agric Food Chem.

